# A distinctive 'microbial signature' in celiac pediatric patients

**DOI:** 10.1186/1471-2180-10-175

**Published:** 2010-06-17

**Authors:** Serena Schippa, Valerio Iebba, Maria Barbato, Giovanni Di Nardo, Valentina Totino, Monica Proietti Checchi, Catia Longhi, Giulia Maiella, Salvatore Cucchiara, Maria Pia Conte

**Affiliations:** 1 Department of Public Health Sciences, 'Sapienza' University of Rome, Piazzale Aldo Moro 5, Rome, 00185, Italy; 2 Department of Pediatrics, "Sapienza" University of Rome, Piazzale Aldo Moro 5, Rome, 00185, Italy

## Abstract

**Background:**

Celiac Disease (CD) is an autoimmune disorder of the small intestine in which dietary gluten ingestion leads to a chronic enteropathy. Recently, scientific evidence suggested a potential role of gut microbiota in CD. To have a snapshot of dominant duodenal microbiota we analyzed the mucosa-associated microbiota of 20 children with CD, before and after a gluten-free diet (GFD) regimen, and of 10 controls. Total DNA was extracted from duodenal biopsies and amplification products of 16S ribosomal DNA were compared by temporal temperature gradient gel electrophoresis (TTGE). TTGE profiles were analyzed by statistical multivariate analysis.

**Results:**

The average number of bands in TTGE profiles was significantly higher (*P *< 0.0001) in active (n.b. 16.7 ± 0.7) and inactive states (n.b. 13.2 ± 0.8) than in controls (n.b. 3.7 ± 1.3). Mean interindividual similarity index was 54.9% ± 14.9% for active disease, 55.6% ± 15.7% for remission state and 21.8% ± 30.16% for controls. Similarity index between celiac children before and after GFD treatment was 63.9% ± 15.8%. Differences in microbiota biodiversity were among active and remission state (*P *= 0.000224) and amid active CD and controls (*P *< 0.001). *Bacteroides vulgatus *and *Escherichia coli *were detected more often in CD patients than in controls (*P *< 0.0001).

**Conclusions:**

Overall, the results highlighted a peculiar microbial TTGE profile and a significant higher biodiversity in CD pediatric patients' duodenal mucosa. The possible pathophysiological role of these microbial differences needs further characterization.

## Background

Celiac disease (CD) is an immune-mediated enteropathy triggered by the ingestion of gluten-containing grains (including wheat, rye, and barley) in genetically susceptible individuals [1]. Its estimated prevalence in Western Countries is near 1% [2]. It is generally agreed that CD is a T-cell mediated disorder in which gliadin derived peptides activate lamina propria T lymphocytes which release proinflammatory cytokines [3]. To date, several peptides including alpha- and gamma-gliadins, have been reported to activate CD4+ lymphocytes via their interaction with HLA-DQ2 and -DQ8 heterodimer on antigen presenting cells (APC) [4].

Recently, scientific evidence showed microecological changes in the intestinal tract of celiac infants, suggesting a potential role of gut microbiota in CD. Alterations in the composition of faecal short-chain fatty acids in CD patients compared with those of healthy controls have been demonstrated [5]. Imbalance in the composition of duodenal microbiota or in faecal bacterial communities of children with CD has also been reported [6-9]. Rod-shaped bacteria have been observed in both gluten-free diet (GFD)-treated and untreated pediatric patients'mucosa, along with a distinctive lectin pattern [10].

The present study was carried out to add further information on the characterization of intestinal microbiota of CD patients, a variable that may represent a new piece of the intriguing puzzle of CD illness. For this purpose we analyzed by TTGE the composition of duodenal mucosa-associated microbiota in the same cohort of GFD untreated and treated CD children and in controls. This prospective study was performed to compare the influence of the disease status on gut microbial composition and to study whether the microbial imbalance could be a peculiar characteristic of the disease.

## Results

### Agglomerative hierarchical classification (AHC)

**Figure 1:**
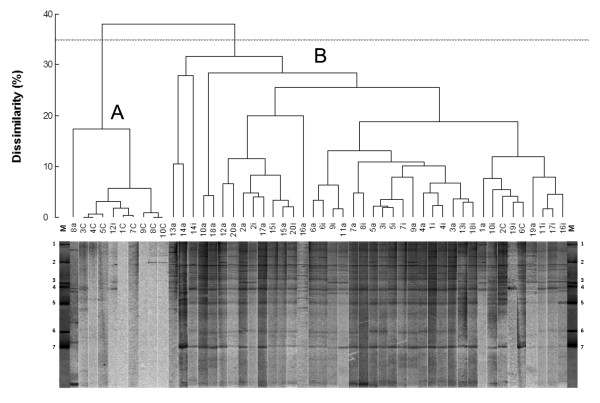
**TTGE profiles dendogram**. TTGE of 16S rDNA amplicons of the bacterial community adherent to duodenal mucosa biopsy samples taken from 20 CD patients who were studied during both active (a) and inactive (i) celiac disease, and 10 controls (c). The dendogram gives a statistically optimal representation of similarities between TTGE profiles based on Euclidean distance dissimilarity matrix and agglomeration method of Ward. The threshold was set at 35% of dissimilarity. Bands of TTGE marker (M) are numbered as follows: 1,6, *Bacteroides vulgatus*; 2,3,7, *Parabacteroides distasonis*; 4, *Bacteroides thetaiotaomicron*; 5, *Escherichia coli*.

The TTGE profiles of PCR amplicons obtained with universal primers were firstly analyzed by XLStat software. The resulting dendrogram presented two major well-defined clusters (fig 1). The first cluster (A) groups 8/10 of control patients, while the second one (B) groups 18/20 of CD patients (Chi-square = 26.51, *P *< 0.005, DF = 1; Fisher's test *P *= 3,46 × 10^-6^). These results highlighted the presence of a dominant microbiota related to the celiac disease, irrespectively to the disease status. The average number of bands in TTGE profiles, calculated by DigiDoc-It software, was significantly higher (*P *< 0.0001) in celiac children (active n.b. 16.7 ± 0.7, inactive n.b. 13.2 ± 0.8) than in controls (n.b. 3.7 ± 1.3), indicating that duodenal mucosa of CD patients showed a higher diversity of associated bacterial population. The average number of bands in TTGE profiles was also significantly higher in active disease than inactive one (*P *= 0.0012). Moreover, interindividual analysis showed a mean Dice similarity index of TTGE profiles of 54.9% ± 14.9% within active disease group, 55.6% ± 15.7% within inactive disease group and 21.8% ± 30.16% within control group. Otherwise, mean Dice similarity index between celiac individuals before and after GFD treatment was 63.9% ± 15.8%.

### Ecological features

**Figure 2:**
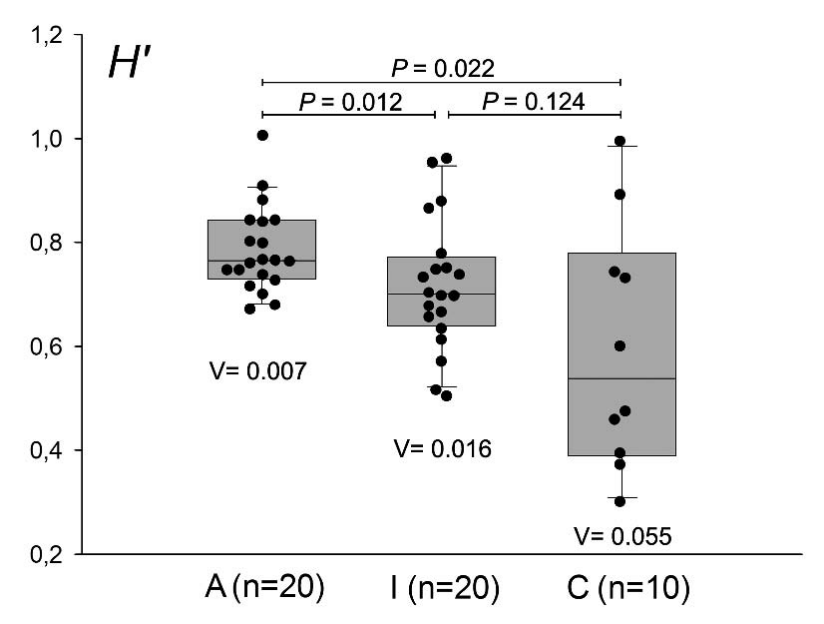
**Duodenal microbial community biodiversity**. Measure of estimated diversity within each biopsy sample obtained from TTGE profiles of CD patients studied during both active (A) and inactive (I) celiac disease, and controls (C). For each group, the diversity index (*H'*), according to Shannon-Weaver and the intra-group variance (V), are shown. The mathematical equation to calculate diversity index for each TTGE profile was with *Pi *= *n_i_*/*N*_tot_, that takes in account the numbers of bands (s), their relative intensity (*n_i_*) and sum (*N*_tot_). *P *values for each inter-group comparison are showed.

Shannon-Wiener index (*H'*) analysis was performed to determine a measure of estimated diversity within each biopsy sample by TTGE profiles. Mean Shannon-Wiener index value differed significantly between active (A) and inactive (I) CD patients, a similar result was obtained between active CD patients and controls. The Shannon-Wiener index among inactive CD patients and controls was not significantly different. (fig 2). The variance values (V) relative to active group revealed a minor data dispersion than inactive and control ones, indicating a more similar microbial biodiversity between its members (fig 2). The carrying capacity of the duodenal system showed mean *Rr *values of: 256.7 ± 98.5, 153.3 ± 64.5, 19.2 ± 41.1 for active, inactive and control group respectively. The mean *Rr *values were highly different among the three groups (p < 0.001).

### Factor discriminating analysis (FDA)

**Figure 3:**
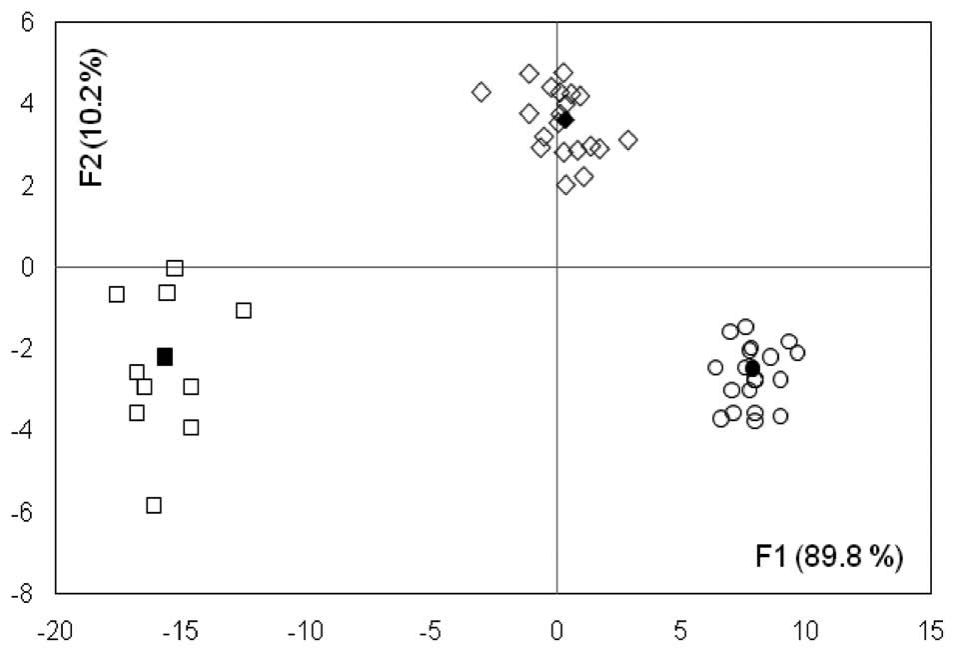
**TTGE profiles FDA model**. Factorial discriminant analysis (FDA) plot for TTGE profiles from CD patients studied, during both active (○) and inactive (◊) celiac disease, and controls (□). The percentages of variation described by the factorial axes (F1,F2) are shown in the parentheses. Center of gravity for each group is reported as filled symbol. Mahalanobis distances (D^2^), between the three centers of gravity were: active vs inactive = 93.030; active vs control = 551.840; inactive vs control = 290.021. Comparison of the aforementioned distances was statistically significant (Mann-Whitney and Wilcoxon tests, *P *< 0.0001) between the three groups of patients. The predictability of the model is 100%.

To improve the analysis of TTGE profiles the more discriminating FDA approach was performed. The Principal Component Analysis (PCA) transformed data showed a well-defined separation between controls, active and inactive celiac groups (*Lambda *= 0.0012, *P *= 0.0044), with a confusion matrix of 0.0% (fig 3). Results from this analysis indicated that the TTGE profiles were sufficient to predict the patient category (active CD, inactive CD or non CD patient) with 100% predictiveness, suggesting the importance of duodenal microbiota in this pathology.

### Partial least square discriminant analysis (PLS-DA)

**Figure 4:**
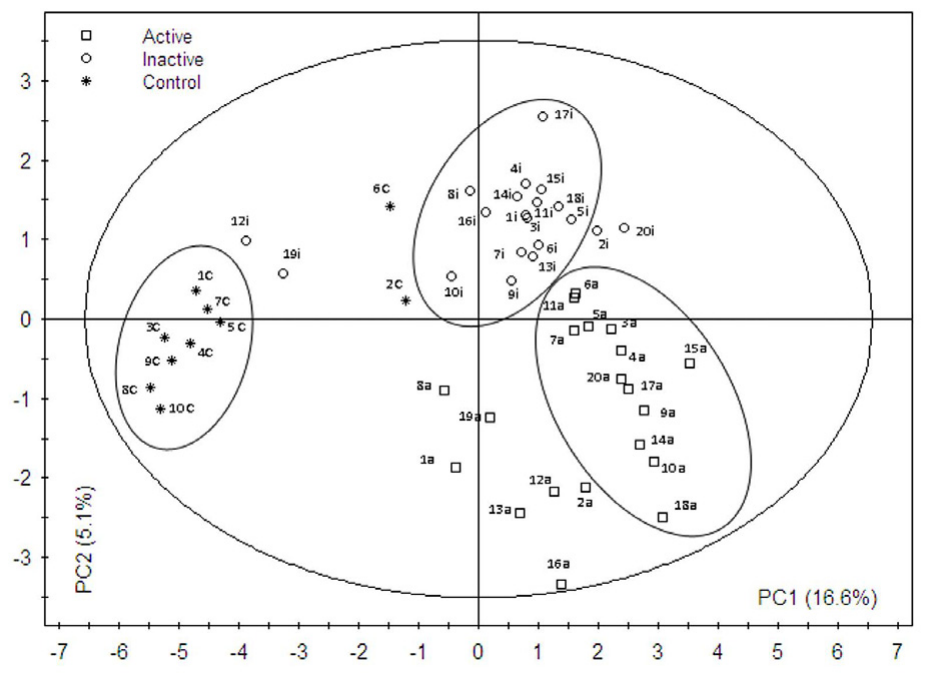
**TTGE profiles PLS-DA model**. PLS-DA score plot of TTGE bands profiles from CD patients, during both active and inactive celiac disease, and controls. In brackets are the percentages of total variation of the dataset explained by the first two components PC1 and PC2. The major ellipse represents Hotelling's T2 range at 95% confidence for the entire dataset (T2_dataset _= 6.51), whilst minor ellipses represent Hotelling's T2 range at 95% confidence for every single group (T2_active _= 2.45, T2_inactive _= 1.88, T2_control _= 1.52). The predictability of PLS-DA model was 88%, with a Fisher's test *P *value of 5.3*10^-8^.

**Figure 5:**
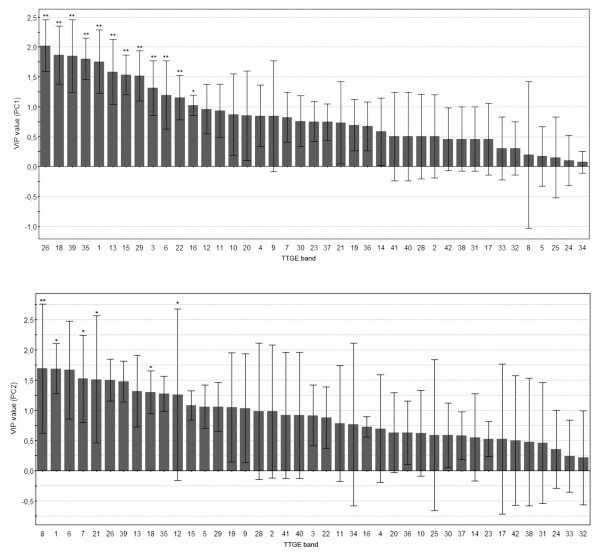
**TTGE band importance**. Hierarchical variable importance (VIP) of discriminatory TTGE bands for PC1 component (partitioning CD/non CD patients, upper panel) and PC2 component (partitioning active CD/in remission CD patients, lower panel). * *P *< 0.05, **P < 0.01.

PLS-DA was employed to investigate peculiar TTGE bands having discriminatory power in separating TTGE profiles in the three groups studied, utilizing the raw data (fig 4). The score plot confirmed a division between the patients' groups. Interestingly, in patients n. 12 and 19 the TTGE profiles of inactive status resulted closer to those of control group. On the basis of PLS-DA score plot, it could be seen that CD patients and controls were separated along Principal Component 1 (PC1) component, whilst active and inactive CD patients were separated along Principal Component 2 (PC2) component. Fig 5 shows hierarchical discriminatory importance of the TTGE bands for PC1 component and PC2 component. The variable importance (VIP) mainly reflected the correlation between the TTGE bands and all the patients groups along a specific principal component axis (PC1 and PC2). The bands with VIP larger than 1 were picked. The TTGE bands picked partitioning CD and non CD-diagnosed patients were: 26, 18, 39, 35, 1, 13, 15, 29, 3, 6, 22, 16. The picked TTGE bands separating active and inactive CD patients were: 8, 1, 6, 7, 21, 26, 39, 13, 18, 35, 12, 15, 5, 29, 19, 9.

### Statistical evaluation of TTGE bands occurrence by PLS-DA

The selected TTGE bands obtained by PLS-DA analysis were statistically evaluated for their occurrence as reported in table 1. The TTGE selected bands (VIP > 1) dividing CD and controls resulted all statistically significant (*P *< 0.05). In the separation between active and inactive CD patients, bands resulted statistically significant were: 8, 1, 7, 21, 18 and 12. Moreover, some of selected TTGE bands run parallel with *E. coli, P. distasonis and B. vulgatus *gel markers used. The parallelism is reported in Tab. 2.

**Table 1:** Statistical importance of discriminating TTGE bands

CD patients vs Controls (PC1)				
**TTGE band §**	**Active + Inactive (%)**	**Control (%)**	**VIP**	***P *value (a)**

26 (*E.coli*)	92.1	20.0	2.023	< 0.0001

18 (*P.distasonis*)	86.8	20.0	1.867	< 0.0001

39 (*P.distasonis*)	89.5	20.0	1.847	0.0001

35	73.7	0.0	1.802	< 0.0001

1 (*B.vulgatus*)	89.5	20.0	1.755	0.001

13	57.9	0.0	1.580	0.000

15	63.2	0.0	1.535	0.001

29	60.5	0.0	1.516	0.001

3	52.6	0.0	1.311	0.003

6	60.5	0.0	1.194	0.010

22	52.6	10.0	1.151	0.007

16	39.5	0.0	1.024	0.018

**Active CD patients vs Inactive CD patients (PC2)**				

**TTGE band§**	**Active(%)**	**Inactive(%)**	**VIP**	***P *****value(b)**

8 (*P.distasonis*)	31.6	0.0	1.691	0.009

1 (*B.vulgatus*)	84.2	94.7	1.687	0.026

6	47.4	73.7	1.667	0.089

7	26.3	0.0	1.522	0.015

21	21.1	0.0	1.507	0.023

26	94.7	89.5	1.498	0.474

39	89.5	89.5	1.475	1.000

13	73.7	42.1	1.316	0.054

18	94.7	78.9	1.299	0.032

35	78.9	68.4	1.271	0.255

12	36.8	10.5	1.258	0.049

15	68.4	57.9	1.079	0.386

5	36.8	15.8	1.056	0.083

29	68.4	52.6	1.054	0.237

19	47.4	63.2	1.046	0.237

9	78.9	94.7	1.031	0.255

§ Bands were self numbered according to the order of appearance (top-bottom) on the TTGE gel and are listed in descending order of importance (VIP) in the PLS-DA model. Between parentheses are reported the species used in the gel marker that run parallel to specific TTGE bands.

(a) Mann-Whitney U-test, α = 0.05

(b) Wilcoxon signed rank test, α = 0.05

**Table 2:** Clinical data of patients' groups

	Celiac Disease	Controls
No. of cases ^(a)^	20	10

Sex ratio (M/F)	8/12	3/7

Age at 1^st ^biopsy^(b)^ (years; median and ranges)	8.3 (1.2-16.1)	11.7 (7.8-20.8)

Weight at birth (Kg) (mean ± SD)	3.3 ± 0.5	3.3 ± 0.4

GFD duration (months; median and ranges)	9 (8-14)	-

Marsh		

IIIA	7/20	-

IIIB	6/20	-

IIIC	7/20	-

(a) None of the patients underwent previous endoscopic assessment

(b) Biopsies were taken in the second part of duodenum

### Species-specific PCR

Results obtained revealed a highly significant difference (P < 0.001) in the prevalence of *B. vulgatus *(85% vs. 20%), and *E. coli *(95% vs. 20%) in CD patients versus controls. A significant difference (P < 0.047) was found in the prevalence of *B. vulgatus *(80% vs. 90%) and in the prevalence (P = 0.039) of *Clostridium coccoides *group (50% vs. 90%) in active CD patients versus inactive CD one. No significant difference was found in the prevalence of *Bifidobacterium spp*. between CD patients and controls (30% vs. 20%, P = 0.742) and between active and inactive CD (20% vs. 40%, P = 0.302).

## Discussion

This is the first longitudinal study on the duodenal mucosa-associated microbiota, carried out on the same cohort of CD pediatric patients (in active and in remission disease), showing a distinctive 'microbial structure' in celiac pediatric patients. The most important results of this study, obtained through multivariate statistical analysis of TTGE profiles, were: i) a dominant duodenal microbiota that could be linked to the disease status (active and remission), outlining differences in the microbiota composition before and after GFD treatment; ii) a significantly higher diversity in dominant microbiota in patients with active disease *vs *the same in remission state, as well as in patients with active disease *vs *controls, as revealed by Shannon-Wiener index. This higher duodenal microbial diversity in CD patients could have a possible harmful impact on the duodenal homeostasis. iii) a higher inter-individual similarity in CD patients than controls, indicating a more homogeneous structure among microbial communities of celiac patients.

Analyzing TTGE profiles, the lowest carrying capacity and the lowest median number of bands found in the duodenal system of the control group can be attributed to an environment particularly adverse or restricted to colonization. The nature of duodenal habitat is radically changed in CD patients, where the carrying capacity and the median number of bands in TTGE profiles are much higher than controls, consequently a thriving colonization could be due to a more habitable environment. It could be speculated that in duodenum the microbial life could be largely inhibited by different factors such the rapid transit of food (transit time 2.5 hours compared to 5 hours of stomach), pancreatic juices or the rapid mucosal turnover. Is therefore likely that a relative small number of definite microbial species or groups are highly adapted to this particular habitat, then the number of TTGE bands found in our control duodenal samples was lower than others found in different intestinal tracts [11,12].

The Dice index analysis of TTGE profiles revealed that mucosa-associated microbiota differed markedly from one patient to another in control group, whereas CD patients showed a high inter-individual similarity. Moreover, before and after GFD treatment, there's a loss of 36.1% of inter-individual similarity. Specifically, the similarity is lost in a homogeneous way between all celiac individuals, as showed by the high similarity Dice index within active and inactive groups. We may speculate that the change in the mucosa lectin patterns both in active and remissive CD, as demonstrated by Forsberg [9], could create more selective microbial adhesive patterns in duodenal mucosa of these patients, promoting a more similar interindividual mucosal colonization.

TTGE bands, having discriminatory power in separating the three patients'groups, have been selected. Some of these TTGE bands run parallel with *E. coli, P. distasonis *and *B. vulgatus *gel markers used. The genera *Bacteroides*, as reported by previous works [8,7], was significantly increased providing a strong correlation between this microbial group and CD [8,6]. Moreover a high prevalence of potentially pro-inflammatory gram negative bacteria was found in the celiac patients' duodenum [6]. Furthermore, the presence of bacteria such *E. coli *and *Bacteroides *spp has been related by other authors [13,14] with mucin degradation and an increase in small intestinal permeability. Although the technique we used does not allow a specific characterization of microbial species or groups of this particular intestinal habitat, it provides a picture of modifications encountered by dominant bacterial groups/species profile of a sample in relation to different factors (i.e. disease status). The presence/absence of bacterial species/groups might act as 'key' or 'regulatory' species leading to a different relative abundance of the present species. To assess this, we need to improve our data by direct sequencing of TTGE bands. TTGE profiles of 18/20 CD patients in remission, with a duodenal histology not fully normalized, clustered together and away from controls. Interestingly, TTGE profiles of 2 CD patients (12 and 19) with a fully histological duodenal normalization at GFD, clustered close to controls as reported by the PLS-DA score plot. This would indicate an association between inflammatory status of intestinal mucosa and the kind of colonizing microbiota. Partial recovery of microbiota composition in the 2 patients with full histological normalization seems to indicate that the mucosa inflammation status is not the only factor driving the kind of microbial composition, but certainly is an influencing factor.

## Conclusions

In conclusion, our data show a potential role of the duodenal microbiota in the CD pathogenesis. Common TTGE profiles in CD patients are probably due to a similar intestinal habitat creating selective pressures that shape a peculiar dominant microbiota. In addition, the occurrence of distinctive TTGE profiles in celiac patients before and after GFD treatment could open new therapeutic strategies aimed at restoring the intestinal ecosystem balance. Further studies will be necessary to develop such strategies, as well as a more accurate identification of bacterial species or groups having discriminatory properties in the CD spectrum.

## Methods

### Patients

Two groups of children referred to the Pediatric Gastroenterology and Liver Unit of the "Sapienza" University of Rome were included in this study: 20 CD (mean age 8.3 years, range 1.2-16.1 years) in active and in remission state (at diagnosis and after at least 9 months of gluten-free diet, respectively) and 10 controls undergoing upper gastrointestinal endoscopy for functional dyspepsia (mean age 11.7 years, range 7.8-20.8 years). The latter tested negative for antitransglutaminase and antiendomysial antibodies with normal IgA levels, while histology of duodenum did not reveal features of CD. Diagnosis of CD had been performed according to ESPGHAN criteria [15]. Table 2 summarizes clinical features of the studied population.

Size appropriate and well oriented endoscopic biopsy specimens were obtained from the second part of the duodenum. The histopathological diagnosis was based on typical mucosal lesions with crypt cell hyperplasia, villous atrophy, and increased number of intra-epithelial lymphocytes (IELs) [16]. All untreated CD patients were positive for antiendomysial and antitransglutaminase antibodies at the time of diagnosis. In all patients there was an endoscopic improvement of duodenal mucosa following gluten withdrawal, but only in 2 of them (patients number 12 and 19) there was also a full histological improvement. None of the children included in the study was treated with antibiotics for at least 3 months before the sampling time. The study protocol was approved by the Committee on Ethical Practice of the 'Policlinico Umberto I' hospital. Children were enrolled in the study after written informed consent from their parents. The biopsy samples were placed in liquid nitrogen immediately after their emission and stored at -80°C until analysis.

### Bacterial strains

The strains listed below were obtained from the American Type Culture Collection (ATCC) and used as marker on TTGE gel electrophoresis: *Bacteroides fragilis *ATCC 23745, *Bacteroides thetaiotaomicron *ATCC 29148, *Bacteroides vulgatus *ATCC 8482, *Parabacteroides distasonis *ATCC 8503, *Escherichia coli *MG1655. Bacterial DNA was extracted with UltraClean kit (MO BIO Laboratories, Solana Beach, California, USA) according to the manufacturer's instructions.

### DNA extraction

Duodenal biopsy specimens from CD and control patients were first quickly washed in 500 μL of physiologic saline with 0.016% dithiothreitol to remove luminal bacteria from the mucus, and then utilized for DNA extraction procedure by DNeasy tissue kit (Qiagen, Hilden, Germany) according to the manufacturer's instructions. In order to obtain maximum yield of both Gram-positive and Gram-negative bacteria, a special step in DNA purification protocol was added, following DNeasy tissue kit manual. Briefly, 180 μL of ATL buffer were added to sample followed by 180 μL volume of enzymatic lysis buffer (20 mM Tris·Cl, pH 8.0, 2 mM sodium EDTA, 1.2% Triton^® ^X-100, lysozyme to 20 mg/ml), and incubated for 30 minutes at 37°C. Next, 25 μL of proteinase K solution and 200 μL of buffer AL were added, followed by an incubation step at 56°C for 30 minutes.

DNA concentration was determined using an Eppendorf biophotometer at 260 nm.

We obtained similar DNA concentrations after kit extraction both from celiac patients and controls biopsies. A Mann-Whitney U test was performed on total DNA concentration (P = 0.11), indicating a similar amount of extracted DNA in both celiac and controls.

### PCR amplification

Polymerase chain reaction (PCR) was performed, as previously described [17] using 400 ng of metagenomic DNA, with minor modification. Briefly, to rule out unspecific PCR products we performed touchdown PCR with a starting annealing temperature of 58°C and decreasing it by 0.5°C each cycle to reach 53°C, then 30 cycles at 53°C were achieved. Same amounts of amplified DNA were also obtained. A Mann-Whitney U test was performed on PCR amplicons (P = 0.23), indicating a similar amount of PCR products in both celiac and controls. To minimize heteroduplex formation and single-stranded DNA (ssDNA) contamination during PCR amplification that might cause sequence heterogeneity in a single TTGE band, an additional 5 cycles of reconditioning PCR was performed, taking 1/10 of the previous PCR volume as template in a new reaction. Moreover, we used 16S rDNA V6-V8 region instead of V3-V4 region that showed coamplification with human DNA. To avoid the problem due to the low bacterial load we performed six individual PCR reactions for each sample. The individual PCR reactions were unified, analyzed by electrophoresis on 2% agarose gels containing ethidium bromide to determine their size (498 bp), and concentrated with SpeedVack (Savant, Holbrook, NY, USA). The unified PCR reactions, before and after the concentration step, were titrated using two different methods: first, densitometry analysis of agarose gel by GelQuest software (Sequentix, Klein Raden, Germany); second, measure of DNA density by biophotometer at 260 nm. The results obtained by such measures were in agreement each other.

PCR protocol was optimized to obtain maximum yield from starting total DNA. The band intensity was quantified at every step (touchdown PCR, reconditioning PCR, concentrated PCR) to ensure an equal DNA concentration. A first-step assessment of DNA suitability for subsequent PCR was achieved through a β-globin gene amplification for each starting sample. Briefly, aliquots of each DNA sample (50 ng) were amplified with specific primers: forward primer, 5'-CAACTTCATCCACGTTCACC-3; reverse primer, 5'-GAAGAGCCAAGGACAGGTAC-3'. Amplification reactions were carried out in a 50-μl volume containing 1× PCR buffer II (Applied Biosystems, Roche, California, USA), 3 mM magnesium chloride, 200 μM each deoxynucleoside triphosphate, 50 pmol each primer and 5 Uμ/l AmpliTaq Gold polymerase (Applied Biosystems). The PCR was carried out under the following conditions: 1 cycle of 95°C for 7 min, 35 cycles of 94°C for 1 min, 55°C for 1 min and 72°C for 1 min and 1 cycle of 72°C for 7 min. 500 ng of DNA of PCR product from each sample were used to perform the subsequent TTGE experiments.

### TTGE analysis of PCR amplicons

We used the DCode Universal mutation detection system (Bio-Rad, Paris, France) for the sequence-specific separation of PCR products. Electrophoresis was performed as previously described [17]. TTGE runs were conducted in triplicate and gel photographed with DigiDoc-It system (UVP, Cambridge, UK).

### Species-specific PCR

We choose to detect those particular species whose presence seems to be involved in celiac disease [7,9]. 16S rDNA gene-targeted primers were utilized to detect them. The primers used were ECO-1 5'-gacctcggtttagttcacaga-3', ECO-2 5'-cacacgctgacgctgacca-3' for *Escherichia coli *(585 bp); BV-1 5'-gcatcatgagtccgcatgttc-3', BV-2 5'-tccatacccgactttattcctt-3' for *Bacteroides vulgatus *(287 bp); g-Ccoc-F 5'-aaatgacggtacctgactaa-3', g-Ccoc-R 5'-ctttgagtttcattcttgcgaa-3' for *Clostridium coccoides *group (438-441 bp), g-Bifid-F 5'-ctcctggaaacgggtgg-3', g-bifid-R 5'-ggtgttcttcccgatatctaca-3' for *Bifidobacterium spp (*549-563 bp). The PCR were performed as previously described [18].

### Data Analysis

*Agglomerative Hierarchical Classification (AHC.) *Dendrogram generated with XLStat 7.5 (Addinsoft, NY, USA) on binary matrix of TTGE variables was evaluated by one-tailed chi-squared test. Data were automatically mean centred and unit variance (UV) scaled. A *P *value equal or less 0.05 was considered statistically significant. Dice similarity index (*S_D_*, mean % ± SD) was calculated within the respective HC and CD groups to assess inter-individual similarity by the formula *S_D _*= (2*n_AB_*)/(*n*A + *n*B), where *n_A _*is the total number of bands in pattern A, *n_B _*is the total number of bands in pattern B and *n_AB _*is the number of bands common to pattern A and B. *Ecological features*. Doc-It LS software (UVP, Cambridge, UK) was used for TTGE bands densitometry peak height quantification, and the correspondent data were analyzed for the microbial biodiversity by Shannon-Wiener index with SigmaPlot 9.0 software. Intra-group variance value (V value) was also calculated. V value defines the variance of data points in each cohort, representing the data dispersion, and indicating the homogeneity/heterogeneity between individuals within a population. In addition, the range-weighted richness (*Rr*), reflecting the carrying capacity of the duodenal system, was calculated by the formula *Rr *= N^2 ^XT_g_, where N is the total number of bands in the TTGE profile and T_g _the temperature gradient comprised between the first and the last band of the same pattern [19]. *Principal Component Analysis (PCA)*. Linearly-dependent TTGE variables were ortogonalized in new factorial axes (F1,F2...F*n*) through PCA by XLStat 7.5 (Addinsoft). The coordinates of the observations on the factorial axes were considered new variables for subsequent factorial discriminant analysis (FDA). *Factor Discriminant Analysis (FDA)*. FDA included in XLStat 7.5 software was performed to create a predictive model useful to classify the patients into one of the three groups according to their TTGE profile. Wilk's Lambda test was used and a *P *value less than or equal to 0.05 was considered statistically significant. *Partial Least Square Discriminant Analysis (PLS-DA)*. PLS-DA included in SIMCA+ software (UMETRICS, Umea, Sweden) was performed to depict score plot of TTGE profiles by means of principal components PC1 and PC2, and to assess TTGE band importance. Data were automatically mean centred and unit variance (UV) scaled by the statistical software. Each TTGE band was hierarchically classified based on a software-assigned variable importance (VIP) value. The variables with VIP value > 1 were chosen as discriminatory. *Non-parametric statistical methods*. For Shannon-Weaver index, species-specific PCR, FDA and PLS-DA, a bilateral Wilcoxon signed rank test was utilized to compare active and inactive CD patients' groups, whilst a bilateral Mann-Whitney U-test was utilized to compare active/inactive CD patients with control group. A *P *value less than or equal to 0.05 was considered statistically significant.

## Competing interests

The authors declare that they have no competing interests.

## Authors' contributions

SS conceived of the study, and participated in its design and coordination and helped to draft the manuscript. VI carried out the TTGE molecular studies, performed the statistical analysis and drafted the manuscript. MB participated in biopsy collection and patients' data. GDN participated in collecting data. VT participated in carrying out TTGE molecular studies. MPC participated in acquisition of data. CL participated in acquisition of data. GM participated in collecting biopsies and patients' data. SC drafted, revised the manuscript and gave final approval to the manuscript. MC helped to draft and revise the manuscript. All authors read and approved the final manuscript.

## References

[B1] Farrell RJ Kelly CP Celiac sprue N Engl J Med 2002 346 180 188 10.1056/NEJMra010852 11796853

[B2] Fortnightly FC Coeliac disease Br Med J 1999 319 236 239 10.1136/bmj.319.7204.236 PMC1116331 10417090

[B3] Ciccocioppo R Di Sabatino A Corazza GR The immune recognition of gluten in coeliac disease Clin Exp Immunol 2005 140 408 416 10.1111/j.1365-2249.2005.02783.x 15932501 PMC1809391

[B4] Qiao SW Bergseng E Molberg Ø Jung G Fleckenstein B Sollid LM Refining the rules of gliadin T cell epitope binding to the disease-associated DQ2 molecule in celiac disease: importance of proline spacing and glutamine deamidation J Immunol 2005 175 254 261 15972656 10.4049/jimmunol.175.1.254

[B5] Tjellstrom B Stenhammar L Hogberg L Fälth-Magnusson K Magnusson KE Midtvedt T Sundqvist T Norin E Gut microflora associated characteristics in children with celiac disease Am J Gastroenterol 2005 100 2784 2788 10.1111/j.1572-0241.2005.00313.x 16393236

[B6] Nadal I Donant E Koninckx CR Calabuig M Sanz Y Imbalance in the composition of the duodenal microbiota of children with coeliac disease Journal of Medical Microbiology 2007 56 1669 1674 10.1099/jmm.0.47410-0 18033837

[B7] Sanz Y Sanchez E Marzotto M Calabuig M Torriani S Dellaglio F Differences in faecal bacterial communities in coeliac and healthy childrens detected by PCR and denaturing gradient gel electrophoresis FEMS Immunol Med Microbiol 2007 51 562 568 10.1111/j.1574-695X.2007.00337.x 17919298

[B8] Collado MC Calabuig M Sanz Y Differences between the Faecal Microbiota of Coeliac Infants and Healthy Controls Curr Issues Intestinal Microbiol 2007 8 9 14 17489434

[B9] Collado MC Donat CE Ribes-Koninckx C Calabuig M Sanz Y Specific duodenal and faecal bacterial groups are associated with pediatric celiac disease J Clin Pathol 2008 62 264 269 10.1136/jcp.2008.061366 18996905

[B10] Forsberg G Fahlgren A Horstedt P Hammarstorm S Hernell O Hammarstorm ML Presence of bacteria and innate immunity of intestinal epithelium in childhood celiac disease Am J Gastroenterol 2004 99 894 904 10.1111/j.1572-0241.2004.04157.x 15128357

[B11] Bik EM Eckburg PB Gill SR Nelson KE Purdom EA Francois F Perez-Perez G Blaser MJ Relman DA Molecular analysis of the bacterial microbiota in the human stomach Proc Natl Acad Sci USA 2006 103 732 737 10.1073/pnas.0506655103 16407106 PMC1334644

[B12] Frank DN St Amand AL Feldman RA Boedeker CE Harpaz N Pace NR Molecular-phylogenetic characterization of microbial community imbalances in human inflammatory bowel diseases Proc Natl Acad Sci USA 2007 104 13780 13785 10.1073/pnas.0706625104 17699621 PMC1959459

[B13] El Asmar R Panigrahi P Bamford P Berti I Not T Coppa GV Catassi C Fasano A Host-dependent zonulin secretion causes the impairment of the small intestine barrier function after bacterial exposure Gastroenterology 2002 123 1607 1615 10.1053/gast.2002.36578 12404235

[B14] Xu J Gordon JI Inaugural Article: Honor thy symbionts Proc Natl Acad Sci USA 2003 100 10452 10459 10.1073/pnas.1734063100 12923294 PMC193582

[B15] Stenhammar L Högberg L Danielsson L Ascher H Dannaeus A Hernell O Ivarsson A Lindberg E Lindquist B Nivenius K How do Swedish pediatric clinics diagnose coeliac disease? Results of a nationwide questionnaire study Acta Pædiatrica 2006 95 1495 1497 10.1080/08035250600636552 17062483

[B16] Marsh MN Studies of intestinal lymphoid tissue. III. Quantitative analyses of epithelial lymphocytes in the small intestine of human control subjects and of patients with celiac sprue Gastroenterology 1980 79 481 492 7429109

[B17] Seksik P Lepage P de la Cochetière MF Bourreille A Sutren M Galmiche JP Doré J Marteau P Search for localized dysbiosis in Crohn's disease ulcerations by temporal temperature gradient gel electrophoresis of 16S rRNA J Clin Microbiol 2005 43 4654 4658 10.1128/JCM.43.9.4654-4658.2005 16145122 PMC1234104

[B18] Conte MP Schippa S Zamboni I Penta M Chiarini F Seganti L Osborn J Falconieri P Borrelli O Cucchiara S Gut-associated bacterial microbiota in pediatric patients with inflammatory bowel disease Gut 2006 55 1760 1767 10.1136/gut.2005.078824 16648155 PMC1856471

[B19] Marzorati M Wittebolle L Boon N Daffonchio D Verstraete W How to get more out of molecular fingerprints: practical tools for microbial ecology Environmental Microbiology 2008 10 1571 1581 18331337 10.1111/j.1462-2920.2008.01572.x

